# Electrochemical Biosensor for Markers of Neurological Esterase Inhibition

**DOI:** 10.3390/bios11110459

**Published:** 2021-11-16

**Authors:** Neda Rafat, Paul Satoh, Robert Mark Worden

**Affiliations:** 1 Department of Chemical Engineering and Materials Science, Michigan State University, 428 S. Shaw Lane, East Lansing, MI 48824, USA; rafatned@msu.edu (N.R.); satoh@msu.edu (P.S.); 2 The Institute for Quantitative Health Science and Engineering, Michigan State University, 775 Woodlot Dr, East Lansing, MI 48824, USA; 3 Department of Biomedical Engineering, Michigan State University, 775 Woodlot Dr, East Lansing, MI 48824, USA

**Keywords:** amperometric biosensor, neural esterase, acetylcholinesterase, inhibition, organophosphate, design, optimization, mathematical model, flux control, dimensionless

## Abstract

A novel, integrated experimental and modeling framework was applied to an inhibition-based bi-enzyme (IBE) electrochemical biosensor to detect acetylcholinesterase (AChE) inhibitors that may trigger neurological diseases. The biosensor was fabricated by co-immobilizing AChE and tyrosinase (Tyr) on the gold working electrode of a screen-printed electrode (SPE) array. The reaction chemistry included a redox-recycle amplification mechanism to improve the biosensor’s current output and sensitivity. A mechanistic mathematical model of the biosensor was used to simulate key diffusion and reaction steps, including diffusion of AChE’s reactant (phenylacetate) and inhibitor, the reaction kinetics of the two enzymes, and electrochemical reaction kinetics at the SPE’s working electrode. The model was validated by showing that it could reproduce a steady-state biosensor current as a function of the inhibitor (PMSF) concentration and unsteady-state dynamics of the biosensor current following the addition of a reactant (phenylacetate) and inhibitor phenylmethylsulfonylfluoride). The model’s utility for characterizing and optimizing biosensor performance was then demonstrated. It was used to calculate the sensitivity of the biosensor’s current output and the redox-recycle amplification factor as a function of experimental variables. It was used to calculate dimensionless Damkohler numbers and current-control coefficients that indicated the degree to which individual diffusion and reaction steps limited the biosensor’s output current. Finally, the model’s utility in designing IBE biosensors and operating conditions that achieve specific performance criteria was discussed.

## 1. Introduction

Electrochemical biosensors are analytical devices that detect analytes by transforming a biochemical reaction into a quantitative, electrical signal. They integrate the specificity of biological recognition molecules (e.g., antibodies) with the advantages of electrochemical detection techniques [1,2]. Electrochemical biosensors benefit from several advantages, such as low cost, ease of use, portability, and simplicity of construction. These advantages make electrochemical biosensors great options for the development of analytical devices in different fields [3,4]. Some of the limitations for electrochemical biosensors are limited shelf life, narrow or limited temperature range for operation, and sometimes high sensitivity of detection results in false-positive results [5]. The electrochemical biosensors can be divided into four major categories based on the electrochemical technique that is used to measure the electrical signal produced by the biochemical mechanism: amperometric biosensors, potentiometric biosensors, conductometric biosensors, and impedimetric biosensors [6].

Amperometric biosensors detect chemicals at a constant electrochemical potential by measuring the oxidation or reduction current produced by electroactive products of a biochemical reaction [7]. Their low cost, high sensitivity, fast response time, simplicity of design, compactness, and potential for miniaturization make amperometric biosensors well suited for detecting a wide range of chemicals and biochemical agents, including disease markers [8,9].

Amperometric biosensors that measure analytes indirectly by their inhibition of target enzymes have been developed for environmental and healthcare applications [10]. Such inhibition-based biosensors can be very sensitive when the target enzyme is inhibited by a very low concentration of its inhibitor [11]. For that reason, significant research has been devoted to developing amperometric biosensors that measure markers of neurological disease processes that inhibit neural esterases, such as acetylcholinesterase (AChE) [12,13,14,15,16,17].

Some organophosphate compounds (OPs) are potent inhibitors of neural esterases and, for that reason, are used as pesticides and as chemical weapons [18]. The well-known neural esterase acetylcholinesterase (AChE) breaks down the neurotransmitter acetylcholine, which chemically relays an impulse across the synapse between two neurons [19]. The inhibition of AChE by OPs prevents acetylcholine hydrolysis, resulting in continuous nerve firing, which can cause severe, acute health issues, including death [20]. Each year, approximately 3 million people are poisoned by organophosphates, accounting for 300,000 deaths worldwide [21].

The gold standard analytical method for OPs is gas/liquid chromatography combined with mass spectroscopy [22]. This method is sensitive, specific, and reliable. However, it is not well suited for many on-site applications because it requires bulky, expensive equipment and involves complicated and time-consuming sample processing by trained technicians [22]. In contrast, inhibition-based amperometric biosensors offer the potential to measure OPs on-site rapidly, inexpensively, with minimal sample processing using a miniature electronic device similar to a personal blood-glucose meter [23].

When an enzyme is strongly inhibited by a specific substance, it may be possible to develop an inhibition-based enzyme biosensor that can specifically detect the presence of the inhibitor in a complex mixture that may include unknown chemicals or pollutants. However, for enzymes that are sensitive to multiple inhibitors, this approach cannot discern which inhibitor(s) is present in the mixture.

Although amperometric biosensors might not offer the same sensitivity and specificity of detection that gas/liquid chromatography-based techniques offer, the fact that they can be developed as portable diagnostic devices for quick and cost-effective initial analysis makes them valuable for initial screening and monitoring. Commercialized miniaturized potentiostats and screen-printed electrodes (SPEs) can be used to develop portable amperometric biosensor systems. SPEs include one or more printed working electrodes, a reference electrode, and a counter electrode printed on a solid substrate. The assay chemistry is performed on the working electrode, and the electrochemical assay is conducted by contacting the SPE with a sample solution. SPEs can replace bulky convention electrochemical cells with a miniaturized system that can be used for simple and quick electrochemical measurements. However, because SPEs are designed to minimize the required space and reagent volume, their measurements are often not as stable and accurate as those conducted in a conventional electrochemical cell.

Research to rationally design and optimize amperometric biosensors for detecting inhibitors of AChE or other neural esterases (e.g., butyrylesterase) has been hampered by the lack of a comprehensive mathematical model able to predict the rates of potentially rate-limiting mass-transfer and chemical reaction steps that produce the amperometric signal. Zhang et al. developed a theoretical model for immobilized-enzyme-inhibition biosensors under the assumption that the inhibition process is diffusion-limited [24]. Choi et al. developed a mathematical model for a fiber-optic biosensor to detect OPs. This model, which simulated both AChE inhibition kinetics and diffusion, was able to optimize the concentrations of AChE and its substrate [25].

We recently developed a novel, integrated experimental and modeling framework that includes a steady-state, mechanistic mathematical model that describes the rate of key mass-transfer and reaction steps and a novel dimensional-analysis approach to assess the degree to which individual mass-transfer and reaction steps limit the biosensor’s amplitude and sensitivity [26]. We then demonstrated the framework’s utility using a novel amperometric electrochemical immunosensor.

In this paper, we apply the framework to an inhibition-based bi-enzyme (IBE) amperometric biosensor assembled on a s SPE. The IBE interface contained a neural esterase (AChE) and an oxidase enzyme (tyrosinase) that generates a redox-reaction loop to amplify the biosensor’s output [27,28,29]. We present a novel, unsteady-state model of the IBE biosensor that consists of unsteady-state mass balance equations describing the mass-transfer and reaction steps that govern the biosensor’s signal. We use experimental results to validate the model and discuss the utility of dimensionless groups based on the model, including current-control coefficients, sensitivity coefficients, and Damkohler numbers, to rationally design and optimize IBE biosensors.

## 2. Materials and Methods

### 2.1. Materials

Sodium phosphate (monobasic and dibasic), AChE (C2888, from Electrophorus electricus), tyrosinase (T3824, from mushroom), bovine serum albumin (BSA), glutaric dialdehyde (50 wt.% solution in water), PMSF, and phenylacetate were obtained from Sigma Aldrich (St. Louis, MO, USA). Ultrapure water (18.2 MΩ) was produced by a Nanopure-UV four-stage purifier (Barnstead International, Dubuque, IA, USA); the purifier was equipped with a UV source and a final 0.2 μm filter. Ultrapure water was used to prepare all aqueous solutions. Screen-printed electrodes were obtained from Conductive Technologies Inc. (New York, NY, USA). and Metrohm DropSens (models DRP-250AT, Asturias, Spain).

### 2.2. Enzyme Electrode Preparation

SPEs were selected as the platform for conducting the experiments for two reasons. First, SPEs are small, inexpensive, and disposable, making them well-suited for POC applications. Second, SPEs are often used in the development of commercialized electrochemical biosensors. SPEs were cleaned by sonication in pure ethanol for 2 min followed by rinsing with ultrapure water. Different types of SPEs, including carbon (DPR-C110), low temperature cured gold (DRP-220BT), high temperature cured gold SPEs (DRP-250AT), and carbon nanotube-modified (DRP-10SWCNT), were tried for the fabrication of the biosensor. The immobilization technique used in this work was based on the crosslinking of two enzymes with glutaraldehyde and bovine serum albumin (BSA) [30]. This technique resulted in an efficient and rapid comobilization of two enzymes. Although this technique worked on all types of SPEs, DPR-250AT resulted in a better repeatability of data. To optimize the immobilization method, a variety of BSA concentrations, glutaraldehyde concentrations, and ratios of AChE to tyrosinase were studied.

To prepare the enzyme solution, 40 µL of 50 mM phosphate buffer pH 7, 20 µL of 20 mg/mL tyrosinase in phosphate buffer, 20 µL of 1 mg/mL AChE in phosphate buffer, 10 µL of 2.7 mg/mL BSA in phosphate buffer, and 10 µL of 4 wt.% glutaraldehyde in water were mixed together just before starting the preparation procedure. To obtain the optimized concentration of the two enzymes, BSA, and glutaraldehyde, 3 µL of enzyme solution (in the case of DropSens SPEs) or 1 µL of enzyme solution (in the case of CTI SPEs) were deposited on the working electrode, and the SPEs were left at 4 °C to dry overnight. The next day, the prepared bi-enzyme-modified SPEs were rinsed with ultrapure water and then stored in phosphate buffer at 4 °C.

### 2.3. PMSF Detection and Electrochemical Measurements

PMSF is used as a model AChE inhibitor because it is less toxic to humans than many OPs. Its reaction mechanism is similar to that of OPs, but its sulfonamide bond with serine’s hydroxyl group in the AChE active site is more stable than a typical OP linkage.

To conduct the electrochemical measurements, the desired electrochemical potential was applied on the SPE’s working electrode relative to the SPE’s printed pseudo-Ag/AgCl reference electrode. The SPE’s counter electrode served as the anode, so the biosensor’s current would not flow through the reference electrode and change its potential. In experiments to detect AChE inhibition by PMSF, 30 µL of 50 mM phosphate buffer (pH 7) was added to the IBE SPE biosensors. A potential of −200 mV relative to an Ag/AgCl reference electrode was maintained on the working electrode using a potentiometer (CHI 660, C.H. Instruments, Austin, TX, USA). An aliquot of phenylacetate solution was added to initiate the IBE’s amperometric signal. Then, after a stable electrochemical signal was obtained, a known amount of PMSF was added while continuously recording the electrochemical current as a function of time. The experiments were repeated in triplicate, and steady-state current values were reported as the mean ± standard deviation of three replicates.

### 2.4. Mechanistic Mathematical Model of the IBE Biosensor Interface

**Figure 1:**
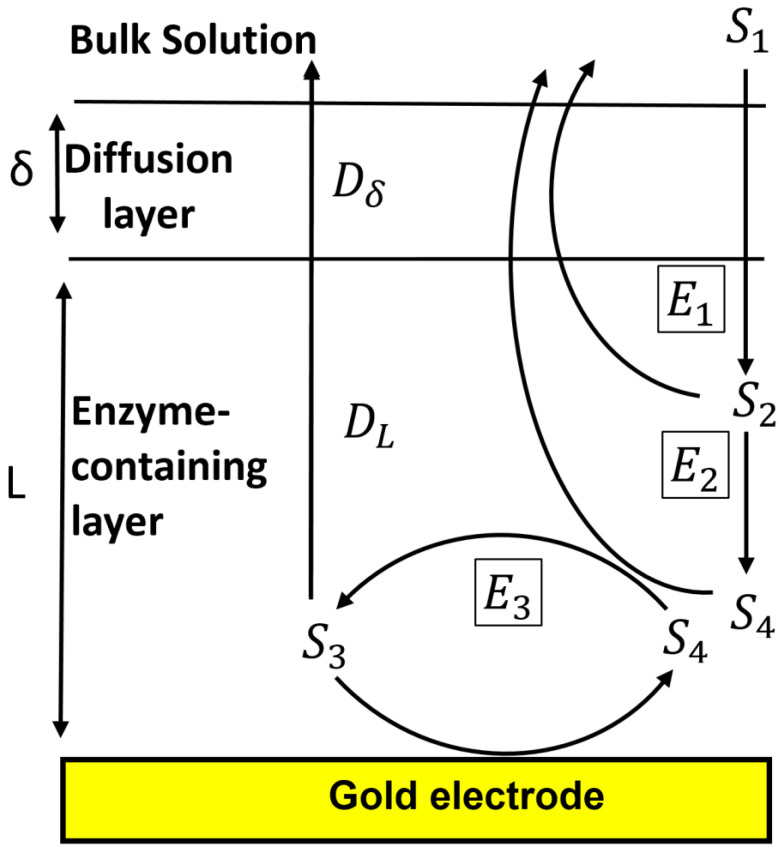
Schematic representation of reactions in the IBE biosensor interface. S_1_, S_2_, S_3_, and S_4_ denote phenylacetate, phenol, catechol, and o-quinone, respectively. E_1_, E_2_, and E_3_ denote acetylcholinesterase, tyrosinase’s phenolase activity, and tyrosinase’s catecholase activity, respectively.

**Figure 2:**
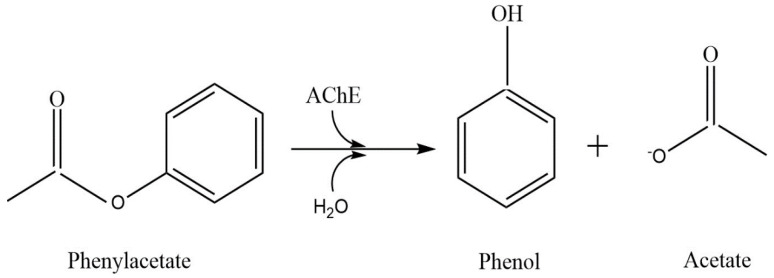
Hydrolysis of phenylacetate with AChE.

The biosensor’s conceptual model (Figure 1) includes a working electrode onto which an enzyme-containing layer of thickness L is bound, a diffusion layer having thickness δ, and the bulk solution. AChE (E_1_) hydrolyzes the reactant phenylacetate (S_1_) to give phenol (S_2_) (Figure 2), which is then oxidized twice by tyrosinase (E_2_)–first to catechol (S_4_) and then to o-quinone (S_3_). The S_4_ can then be reduced back to S_3_ at the electrode, generating the biosensor’s output current. This current is amplified by a redox-recycle loop in which each molecule of S_4_ produced by the combined actions of E_1_ and E_2_ may be sequentially oxidized by E_2_ and then reduced at the electrode many times, with additional current being produced in each cycle.

**Figure 3:**
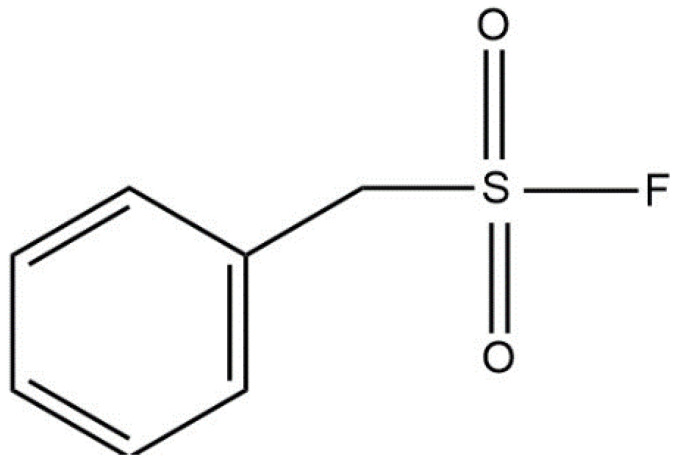
Molecular structure of PMSF.

The concentration of PMSF (I, Figure 3) in a sample was determined from the drop in the biosensor’s current following addition and exposure to PMSF.

The biosensor’s mathematical model consists of a set of coupled, unsteady-state, differential mass-balance equations that take into account: (1) the rate of mass transfer of phenylacetate, phenol, catechol, O-quinone, and PMSF in the x-direction through the diffusion layer (L < x < L + δ) and the enzyme-containing layer (0 < x < L); (2) the kinetics of the enzyme-catalyzed chemical reactions by AChE and tyrosinase within the enzyme-containing layer; and (3) the kinetics of electrochemical reduction of o-quinone at the gold electrode. The enzymes’ concentrations are assumed to be uniform across the enzyme-containing layer [31,32]. The bulk solution is assumed to be well-mixed, with the concentrations of all chemical species remaining constant at their initial values [33]. The PMSF bulk concentration is assumed to be zero before the addition time ($t=T_{0}$).

#### 2.4.1. AChE Inactivation and Enzyme Kinetics

PMSF inhibits AChE’s reaction rate (E_1_) by binding at AChE’s active site [34]. The sulfonyl group of PMSF (Figure 3) mimics the carbonyl group of phenylacetate’s transition state. As a result, the hydroxyl group of the serine residue in AChE’s active site nucleophilically attacks the sulfonyl group, which can lead to irreversible, covalent sulfonylation of AChE [35]. In this model, we assumed that the rate of PMSF (I) consumption is equal to the rate of AChE inactivation.

**Figure 4:**
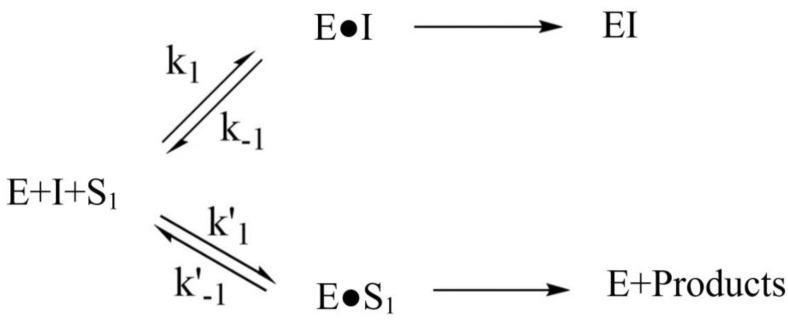
Inhibition mechanism of AChE (E) with PMSF (I) in the presence of substrate (S_1_).

The general scheme for inactivation of AChE with PMSF (I) in the presence of the substrate (S_1_) is shown in Figure 4.

Studies have shown that AChE inhibition with PMSF follows pseudo-first-order kinetics [35] (Equation (1)):

(1) $$\ln\;\frac{V_{\max,1}^{\prime }}{V_{\max,1}}=-k^{\prime }t^{\prime }$$

where $V_{\max,1}$ and $V_{\max,1}^{\prime }$ are the maximum reaction rates for AChE in the absence of the inhibitor and when incubated with inhibitor for a time of $t^{\prime }$, respectively. $k^{\prime }$ is the pseudo-first-order rate constant for the inactivation of AChE by PMSF (Equation (2)):

(2) $$\;k^{\prime }=\frac{k_{2}\left[I\right]}{\frac{1}{\left(1-\gamma \right)}k_{I}+\left[I\right]}$$

The affinity of PMSF for AChE is given by the Michaelis–Menten type constant, $k_{I}$ [35]:

(3) $$k_{I}=\frac{k_{-1}+k_{2}}{k_{+1}}$$

where $k_{+1}$ and $k_{-1}$ are the forward and backward rate constants for the formation of the Michaelis–Menten type complex, and $k_{2}$ is the sulfonylation rate constant (Figure 4). The value of γ is given by Equation (4), where $K_{m,1}$ is the Michaelis–Menten constant for phenylacetate hydrolysis.

(4) $$\gamma =\frac{\left[S_{1}\right]}{\left[S_{1}\right]+K_{m,1}}$$

PMSF competes with phenylacetate for the active site of AChE, thereby changing $K_{m,1}$ to an the apparent value $K_{m,1}^{\prime }$ (Equation (5)) [25].

(5) $$K_{m,1}^{\prime }=K_{m,1}\left(1+\frac{\left[I\right]}{k_{I}}\right)$$

Equations (6)–(8) describe the enzymatic kinetics of AChE in the presence of PMSF, where $k_{\mathrm{cat},1}$ is the turnover number of AChE for phenylacetate. By assuming that the rate of PMSF (I) consumption equals the rate of enzyme inactivation, Equation (9) was derived to describe the rate of PMSF (I) consumption.

(6) $$v_{1}=\frac{V_{\max,1}^{\prime }[S_{1}]}{K_{m,1}^{\prime }+[S_{1}]}$$

(7) $$V_{\max,1}^{\prime }=V_{\max,1}e^{-k\prime t}$$

(8) $$V_{\max,1}=k_{\mathrm{cat},1}E_{1}$$

(9) $$\frac{dI}{dt}=-k^{\prime }E_{1}e^{-k^{\prime }t}$$

#### 2.4.2. Tyrosinase Enzyme Kinetics

**Figure 5:**
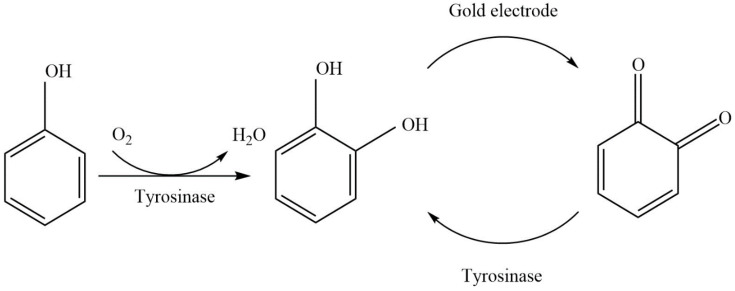
Scheme of phenol oxidation with tyrosinase to produce o-quinone.

Tyrosinase exhibits two enzymatic activities: monophenolase activity, which catalyzes the hydroxylation of phenol to produce o-diphenol (catechol) and catecholase activity, which catalyzes the oxidation of catechol to o-quinone. Figure 5 shows the scheme for the two-step oxidation of phenol with tyrosinase.

Studies have shown that the hydroxylation step (monophenolase activity) is much slower than the oxidation step (catecholase activity), and therefore limits the o-quinone production rate [36]. Therefore, we assumed that the rate $(v_{2})\;$of o-quinone (S_4_) production from phenol (S_2_) can be obtained from Equations (11)–(12), where E_2_ corresponds to phenolase activity of tyrosinase. The rate $(v_{3})\;$of conversion of catechol (S_3_) to o-quinone (S_4_) can be given by Equations (12) and (13), where E_3_ denotes the catecholase activity of tyrosinase [29]

(10) $$v_{2}=\frac{V_{\max,2}[S_{2}]}{K_{m,2}+[S_{2}]}$$

(11) $$V_{\max,2\;}=k_{\mathrm{cat},2}\;E_{2}$$

(12) $$v_{3}=\frac{V_{\max,3}[S_{3}]}{K_{m,3}+[S_{3}]}$$

(13) $$V_{\max,3\;}=k_{\mathrm{cat},3}\;E_{3}$$

The molecules of o-quinone produced by tyrosinase are assumed to be reduced back to catechol at the working electrode at a rate described by the Butler–Volmer equation (Equation (14)):

(14) $$J={\mathrm{nFD}}_{L}{\left[\frac{\partial Q}{\partial x}\right]}_{x=0}={\;\mathrm{nFK}}_{0}{\left[Q\right]}_{x=0}{\;e}^{\left(-\frac{\alpha \mathrm{nF}\left(E-E_{h}\right)}{\mathrm{RT}}\right)}-{\mathrm{nFK}}_{0}{\left[C\right]}_{x=0}{\;e}^{\left(\frac{\left(1-\alpha \right)\mathrm{nF}\left(E-E_{h}\right)}{\mathrm{RT}}\right)}$$

where J is the electric current density, n is the number of electrons transferred (e.g., n = 2 for the electrochemical reduction of Q), α is the charge transfer coefficient (assumed to be 0.4), F is the Faraday constant (96,485 C mol^−1^), $K_{0}$ is the apparent electron transfer rate constant for Q, R is the universal gas constant (8.314 J K^−1^ mol^−1^), T is the absolute temperature (298 K), and $E_{h}\;$is the redox potential for electrochemical reduction of Q to C under the experimental conditions. An $E_{h}$ value of 0.15 V was determined as the midpoint between the cathodic peak and anodic peak of cyclic voltammogram obtained under the same conditions.

#### 2.4.3. Mass Balance Equations

The mass balance equations including diffusion and enzymatic reaction for S_1_, S_2_, S_3_, S_4_, and I across the enzyme-containing layer (0 < x < L) can be derived (Equations (15)–(19)).

(15) $$\frac{\partial S_{1}}{\partial t}=D_{L}\frac{\partial^{2}S_{1}}{\partial x^{2}}-\frac{V_{\max,1}^{\prime }[S_{1}]}{K_{m,1}^{\prime }+[S_{1}]}$$

(16) $$\frac{\partial S_{2}}{\partial t}=D_{L}\frac{\partial^{2}S_{2}}{\partial x^{2}}-\frac{V_{\max,2}E_{2}[S_{2}]}{K_{m,2}+[S_{2}]}+\frac{V_{\max}^{\prime }[S_{1}]}{K_{m,1}^{\prime }+[S_{1}]}$$

(17) $$\frac{\partial S_{3}}{\partial t}=D_{L}\frac{\partial^{2}S_{3}}{\partial x^{2}}-\frac{V_{\max,3}E_{3}[S_{3}]}{K_{m,3}+[S_{3}]}$$

(18) $$\frac{\partial S_{4}}{\partial t}=D_{L}\frac{\partial^{2}S_{4}}{\partial x^{2}}+\frac{V_{\max,3}[S_{3}]}{K_{m,3}+[S_{3}]}$$

(19) $$\frac{\partial I}{\partial t}=D_{L}\frac{\partial^{2}I}{\partial x^{2}}+{k^{\prime }E}_{1}e^{-k^{\prime }t}$$

#### 2.4.4. Boundary Conditions

Because Q reduction at the electrode generates C in equimolar amounts, the fluxes of Q and C at x = 0 were assumed to be equal in magnitude but opposite in sign (Equation (20)).

(20) $$D_{L}{\left[\frac{\partial S_{4}}{\partial x}\right]}_{x=0}=-D_{L}{\left[\frac{\partial S_{3}}{\partial x}\right]}_{x=0}$$

At x = 0, S_1_, S_2_, S_3_, and I are assumed not to be consumed or produced at the electrode (Equation (21)):

(21) $${\left[\frac{\partial S_{1}}{\partial x}\right]}_{x=0}=0,{\left[\frac{\partial S_{2}}{\partial x}\right]}_{x=0}=0,{\left[\frac{\partial S_{3}}{\partial x}\right]}_{x=0}=0,\;{\left[\frac{\partial I}{\partial x}\right]}_{x=0}=0$$

Partitioning kinetics of all reactants were assumed to be rapid enough that the interfacial concentrations at the boundaries of the diffusion layer and enzyme-containing layer remained at equilibrium. Identical partition coefficients ($k_{p}=1)\;$were assumed for all reactants (Equations (22)–(26)).

(22) $${\left[S_{1}\right]}_{L+}=k_{p}{\left[S_{1}\right]}_{L+}$$

(23) $${\left[S_{2}\right]}_{L+}=k_{p}{\left[S_{2}\right]}_{L+}$$

(24) $${\left[S_{3}\right]}_{L+}=k_{p}{\left[S_{3}\right]}_{L+}$$

(25) $${\left[S_{4}\right]}_{L+}=k_{p}{\left[S_{4}\right]}_{L+}$$

(26) $${\left[I\right]}_{L+}=k_{p}{\left[S_{4}\right]}_{L+}$$

The bulk solution (where x = ∞) contained S_1_ at a concentration of S_1_(∞) but negligible concentrations of S_2_, S_3_, and S_4_ (Equations (27)–(31)).

(27) $${\left[S_{1}\right]}_{x=\infty }=C\left(\infty \right)$$

(28) $${\left[S_{2}\right]}_{x=\infty }=0$$

(29) $${\left[S_{3}\right]}_{x=\infty }=0$$

(30) $${\left[S_{4}\right]}_{x=\infty }=0$$

(31) $${\left[I\right]}_{x=\infty ,\;t<T0}=0,\;{\left[I\right]}_{x=\infty ,\;T0\;<\;t}=I\left(\infty \right)$$

Because no reaction is assumed to occur in the diffusion layer, the flux of species entering this layer was assumed to equal that exiting it (Equations (32)–(36)).

(32) $$D_{L}{\left[\frac{\partial S_{1}}{\partial x}\right]}_{x=L-}=\frac{D_{\delta }}{k_{p}\;\delta }\{k_{p}{\;S}_{1}\left(\infty \right)-{\left[S_{1}\right]}_{x=L-}\}$$

(33) $$D_{L}{\left[\frac{\partial S_{2}}{\partial x}\right]}_{x=L-}=-\frac{D_{\delta }}{\;\delta }\{{\left[S_{2}\right]}_{x=L+}-0\}=-\frac{D_{\delta }}{k_{p}\;\delta }{\left[S_{2}\right]}_{x=L-}$$

(34) $$D_{L}{\left[\frac{\partial S_{3}}{\partial x}\right]}_{x=L-}=-\frac{D_{\delta }}{\;\delta }\{{\left[S_{3}\right]}_{x=L+}-0\}=-\frac{D_{\delta }}{k_{p}\;\delta }{\left[S_{3}\right]}_{x=L-}$$

(35) $$D_{L}{\left[\frac{\partial S_{4}}{\partial x}\right]}_{x=L-}=-\frac{D_{\delta }}{\;\delta }\{{\left[S_{4}\right]}_{x=L+}-0\}=-\frac{D_{\delta }}{k_{p}\;\delta }{\left[S_{4}\right]}_{x=L-}$$

(36) $$D_{L}{\left[\frac{\partial I}{\partial x}\right]}_{x=L-}=\frac{D_{\delta }}{k_{p}\;\delta }\{k_{p}\;I\left(\infty \right)-{\left[I\right]}_{x=L-}\}$$

#### 2.4.5. Initial Conditions

Initial conditions of phenylacetate at injection time (t = 0) are given in Equation (37):

(37) $${\left[S_{i}\right]}_{\;i=2:4,0⩽x⩽L}=0,\;{\left[S_{1}\right]}_{0⩽x<L}=0,{\left[S_{1}\right]}_{x=L}=S_{1}\left(\infty \right)$$

The inhibitor concentration (I) was assumed to be zero before it was injected at ($T_{0})$ and was assumed to be constant throughout the enzyme-containing layer and solution afterward (Equation (38)):

(38) $${\left[I\right]}_{0<t<T_{0},}=0,\;{\left[I\right]}_{T_{0}<t}=I\left(\infty \right)$$

A splitting-finite-difference algorithm was programmed in MATLAB and used to solve the mass balance equations (Equations (15)–(19)) numerically using the parameters given in Table 1 and the boundary and initial conditions given in Equations (20)–(38).

## 3. Results and Discussion

### 3.1. Biosensor’s Response to PMSF

**Figure 6:**
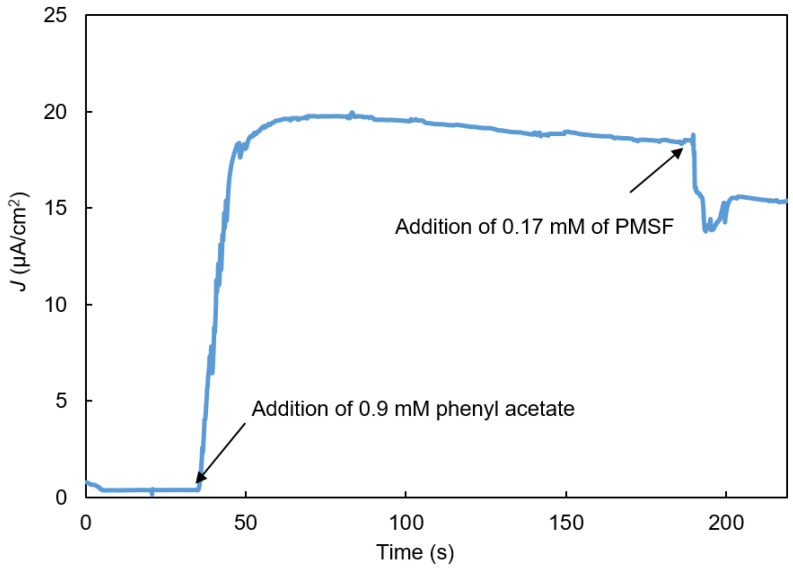
Current vs. time response of the bi-enzyme biosensor to the addition of phenylacetate (S_1_) to obtain a final phenylacetate (S_1_) concentration of 0.9 mM followed by the addition of inhibitor PMSF to obtain a final PMSF concentration of 0.17 mM.

Figure 6 shows a typical amperometry experiment to detect PMSF. Phenylacetate was added at about 35 s, and PMSF was added at about 190 s. As soon as a buffer sample containing PMSF was added, the biosensor’s current rapidly declined and then returned to a relatively stable value whose magnitude varied with the PMSF concentration in the sample. A first-order time constant for the IBE biosensors’ response to PMSF (defined as 63% toward the relatively stable current value) was typically about 20 s.

**Figure 7:**
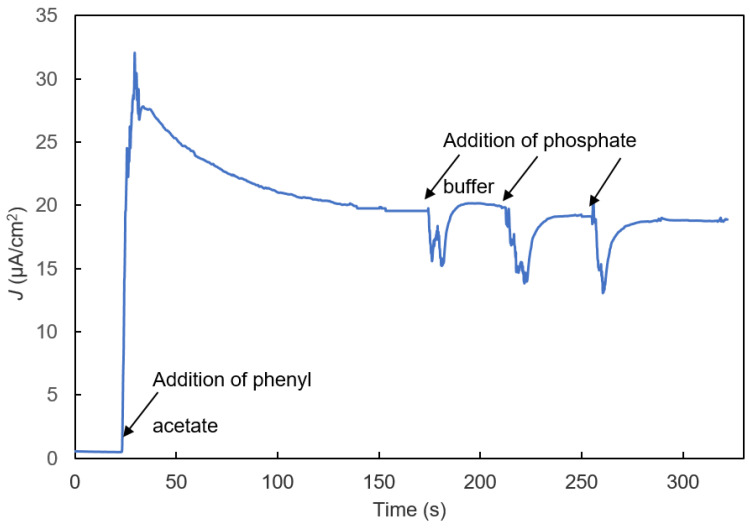
Control experiment to study the effect of phosphate buffer addition on the bi-enzyme biosensor’s signal.

Control experiments were conducted to characterize the IBE biosensor’s response to blank samples with the addition of a bolus of the buffer without PMSF (Figure 7). As soon as a buffer sample without PMSF was added, the biosensor’s signal rapidly declined and then returned to a relatively stable current very close to that before the blank sample was added.

**Figure 8:**
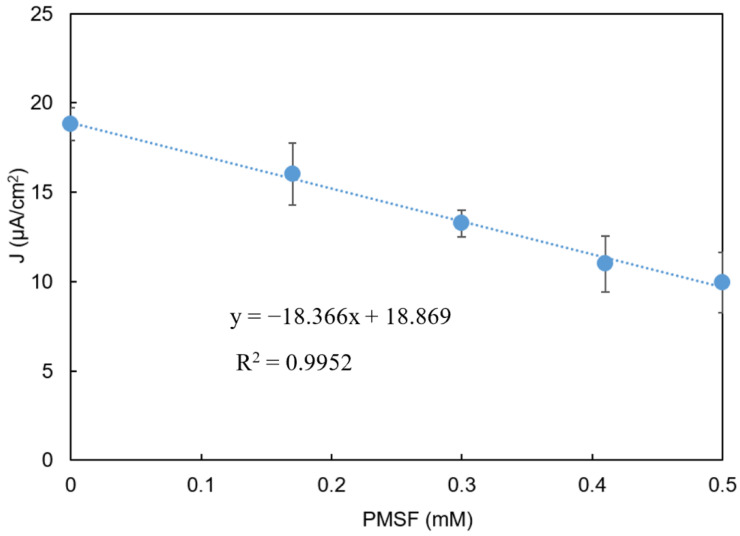
Current vs. PMSF concentration. Error bars indicate the mean ± standard deviation of three replicates. Phenylacetate: 0.9 mM. y = −18.366x + 18.869, R^2^ = 0.9952.

The PMSF-challenge experiments described above were repeated for samples containing a variety of PMSF concentrations. Figure 8 shows the current to which the signal returned after the initial decline as a function of the PMSF concentration in the sample. The calibration curve had a sensitivity (slope) of 18.4 μA cm^−2^ (mM PMSF)^−1^ and an R^2^ value for a linear fit of 0.995.

### 3.2. Validation of the Mathematical Model and Simulation of the Biosensor’s Response

The numerical model successfully simulated the biosensor’s behavior shown in Figure 6, Figure 7 and Figure 8. To explain the initial, rapid signal decline triggered by sample addition, we hypothesized that the increase in convective mass transfer while the sample was being pipetted into the solution on the SPE altered the pseudo-steady-state concentration gradients of the reacting chemical intermediates (S_2_, S_3_, and S_4_) in the enzyme-containing layer. To simulate this effect in the model, we decreased the concentrations of these intermediates in the enzyme-containing layer by some fraction (e.g., 20%) at t = T_0_ (Equations (39)–(41)):

(39) $${\left[S_{2}\right]}_{0<x<L,\;t=T_{0}^{+}}=0.8\;{\left[S_{2}\right]}_{0<x<L,\;t=T_{0}^{-}}$$

(40) $${\left[S_{3}\right]}_{0<x<L,\;t=T_{0}^{+}}=0.8\;{\left[S_{3}\right]}_{0<x<L,\;t=T_{0}^{-}}$$

(41) $${\left[S_{4}\right]}_{0<x<L,\;t=T_{0}^{+}}=0.8\;{\left[S_{4}\right]}_{0<x<L,\;t=T_{0}^{-}}$$

**Figure 9:**
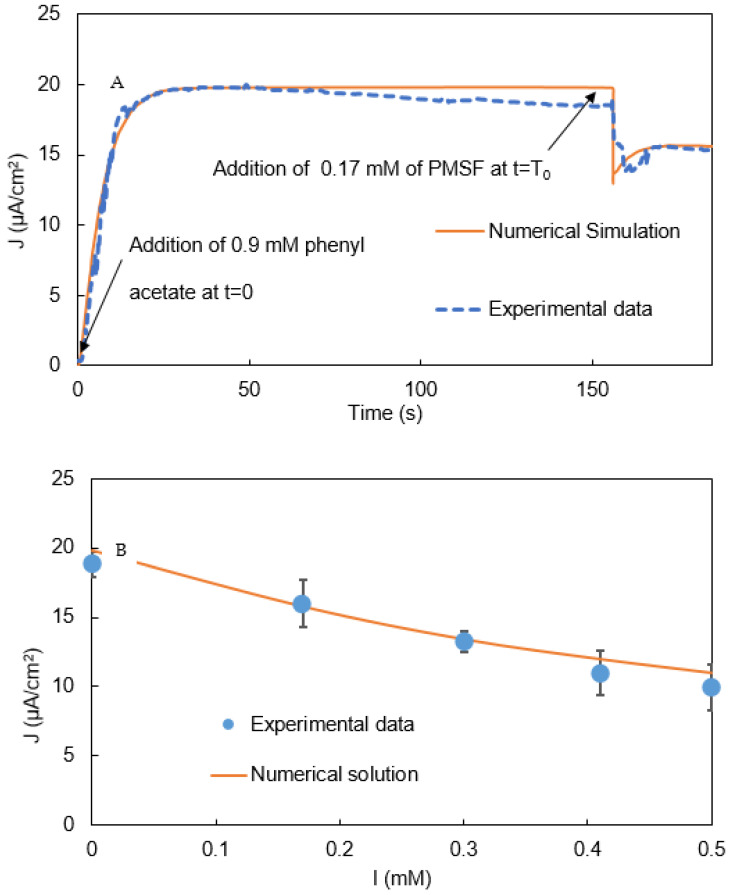
(**A**): Simulated the bi-enzyme biosensor’s signal vs. time. (**B**): Simulated current density vs. PMSF concentration (I). Error bars indicate the mean ± standard deviation of three replicates.

This change enabled the model to predict the biosensors observed dynamics following sample addition, including the sudden drop in the biosensor’s signal, followed by a return to a stable current (Figure 9A), providing support for the hypothesis.

The model was also able to accurately predict the relatively stable current that resulted after the initial biosensor-response dynamics as a function of the PMSF concentration in the sample (Figure 9B).

Figure 6 and Figure 7, and Figure 9B show that after phenylacetate was added, the biosensor’s current increased rapidly, went through a maximum, and then exhibited a gradual decay. The mechanism responsible for the decay is unknown, but it may result from the formation of byproducts of o-quinone reduction at the electrode that are not re-oxidized as rapidly by tyrosinase as catechol. The result of such a reaction would be a gradual increase in the byproduct concentration, and decrease in the catechol and o-quinone concentrations, and, consequently, a gradual decrease in the biosensor’s current.

#### Signal Amplification by Redox-Recycle Loop

The degree of biosensor signal amplification due to the redox-recycle loop involving catechol and O-quinone (Figure 5) activity can be quantified using an amplification factor (AF), which is defined as the ratio of the biosensor’s current density (J) in the presence of catecholase activity to that in the absence of the catecholase activity (Equation (42)) [29]:

(42) $$\mathrm{AF}=\frac{{J|}_{E_{3}\neq 0}}{{J|}_{E_{3}=0}}$$

**Figure 10:**
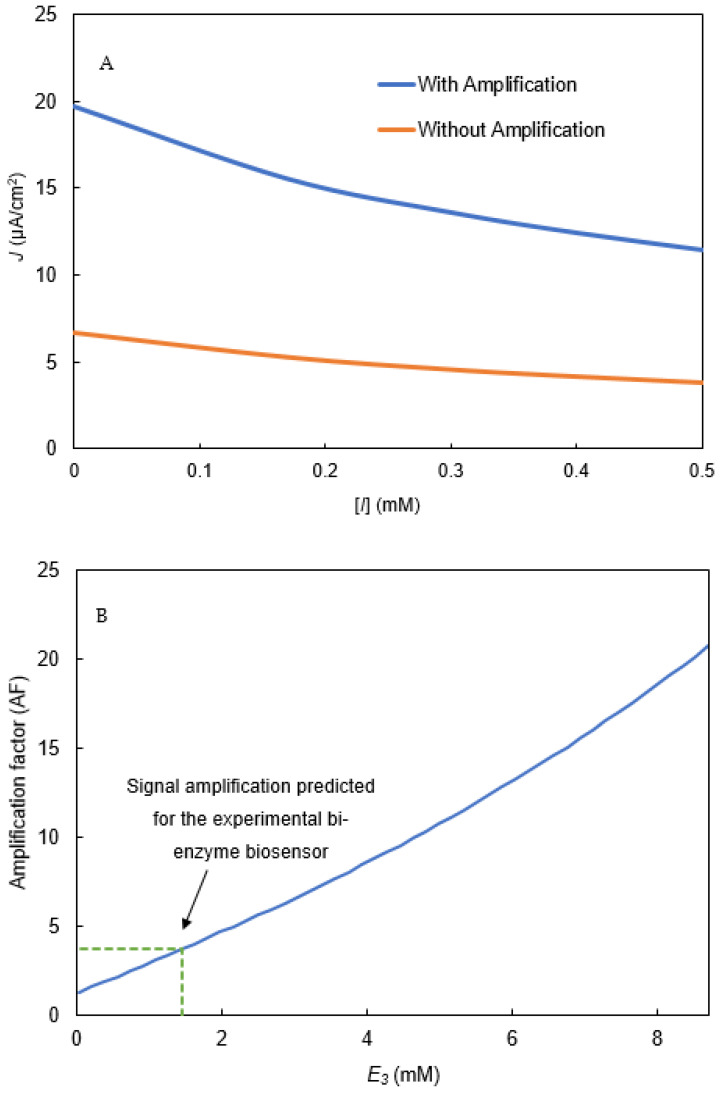
(**A**): Simulated current density with and without (E_3_ = 0) the amplification system in the bi-enzyme biosensor. (**B**): Signal amplification in bi-enzyme biosensor due to S_3_ recycling caused by catecholase activity (E_3_).

After validating the biosensor model (Figure 9A,B), we used it to explore the extent of signal amplification under a variety of operating conditions. Figure 10A shows the model-predicted output current both in the presence and absence of the amplification system. To predict the absence of amplification system zero, catecholase activity of tyrosinase was set to zero in the model. The predicted AF of about three across the range of [I] simulated indicates that redox amplification increases the biosensor’s current roughly three-fold under the experimental conditions.

The effect of catecholase concentration (E3) on the predicted AF was also explored with the model (Figure 10B). This result shows an increasing tyrosinase concentration would increase the biosensor’s output by increasing the redox amplification.

### 3.3. Biosensor Sensitivity

Biosensor sensitivity (S) with respect to PMSF concentration [I] is defined in Equation (43):

(43) $$S=\frac{dJ}{d\left[I\right]}$$

**Figure 11:**
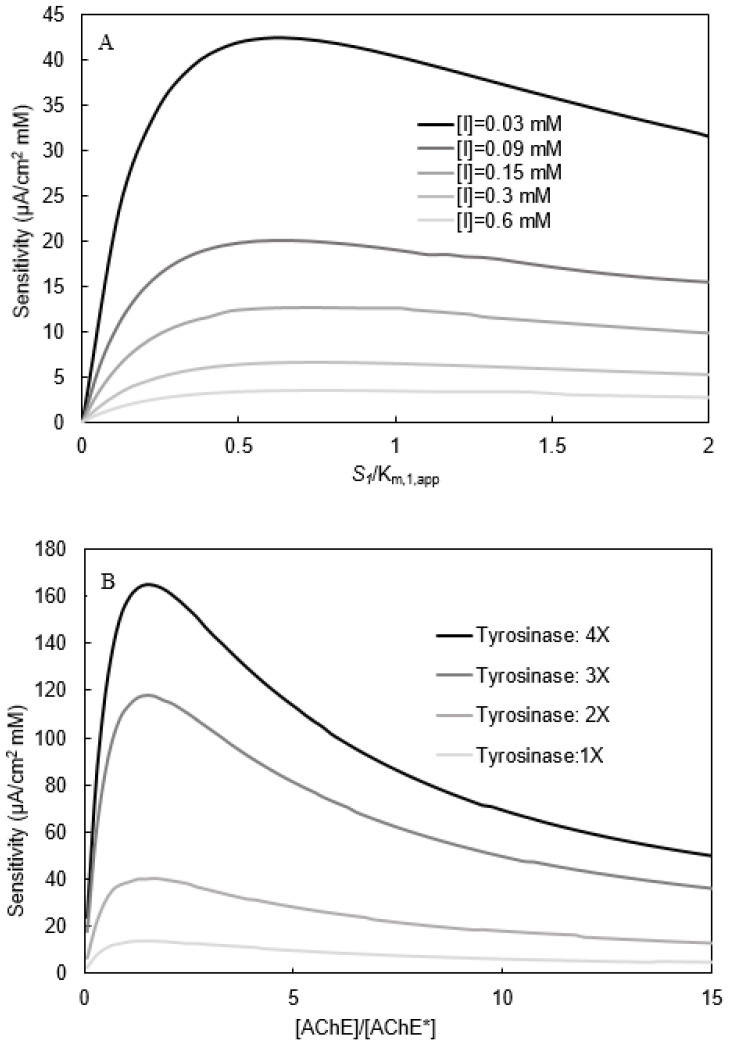
(**A**): Sensitivity vs. phenylacetate concentration (S_1_). S_1_ has been normalized with K_m,1,app_. (**B**): Sensitivity vs. [AChE] at different tyrosinase concentrations. [AChE] has been normalized with [AChE*] = 3 µM. [I] = 0.3 mM.

To calculate S for a given set of experimental conditions, the incremental change in J (∆J) resulting from an incremental change in [I] (∆[I]) was calculated by the model. Then the asymptotic value of the ratio ∆J/∆[I] as ∆[I] approached zero was determined and used as the S value for those conditions. The resulting S values were plotted as function of a dimensionless phenylacetate concentration ([S_1_]/K_m,1,app_) for several [I] values (Figure 11A).

All the sensitivity curves exhibited a maximum value for the following reasons. At low [S_1_]/K_m,1,app_ values, the S_1_ hydrolysis rate, and thus the J value, is so low that the maximum possible drop in J due to an increase on PMSF concentration is also small. At large [S_1_]/K_m,1,app_ values, almost all AChE active sites are occupied with S_1_ values, and are unavailable to bind to PMSF molecules; thus the addition of PMSF has little effect on J.

For all [S_1_]/K_m,1,app_ values shown, sensitivity values increased as the PMSF concentration decreased. The lower the PMSF concentration, the higher the S_1_ hydrolysis rate, the J value, and the maximum possible drop in J as the PMSF concentration is increased.

The calculated S values were also plotted as a function of dimensionless AChE concentration for several dimensionless tyrosinase concentrations (Figure 11B).

For all tyrosinase concentrations, plots of sensitivity vs. (AChE)/(AChE*) exhibited a maximum. At low [AChE]/[AChE*] values, they are equal to the S_1_ hydrolysis rate, and thus the J value is so low that the [AChE]/[AChE*] values are different from the S_1_ hydrolysis limits, J; therefore, the inhibition of AChE by PMSF addition has little effect on J.

For all of the [AChE]/[AChE*] values shown, sensitivity values increased as the tyrosinase concentration increased. This effect is attributed to the catecholase activity, and thus greater amplification and J values that occur at higher tyrosinase concentrations (Figure 11B).

### 3.4. Identification of Rate Limiting Step

In a recent publication, we described the use of dimensionless groups to assess the rate-limiting step(s) in amperometric biosensors. The biosensor’s current results from the interplay of multiple mass transfer and reaction steps, each of which has the potential to be rate-limiting (i.e., control the biosensor current output) to some extent. Because the mechanistic model predicts the rate of each step, it enables the extent to which each step is rate-limiting to be calculated.

We used Equation (44) and parameter values from Table 1 to calculate the Damkohler number (σ), defined as the square root of the dimensionless ratio of the relative rates of enzymatic reaction ($\frac{V_{\max}}{K_{M}}$) and diffusional mass transfer ($\frac{D_{L}}{L^{2}})$ within the enzyme-containing layer [37].

(44) $$\sigma^{2}=\frac{V_{\max}L^{2}}{D_{L}K_{M}}$$

The σ values for AChE and tyrosinase were in the order of 10^−5^, indicating that the diffusion steps are many orders of magnitude faster than the reaction steps [32,38].

**Figure 12:**
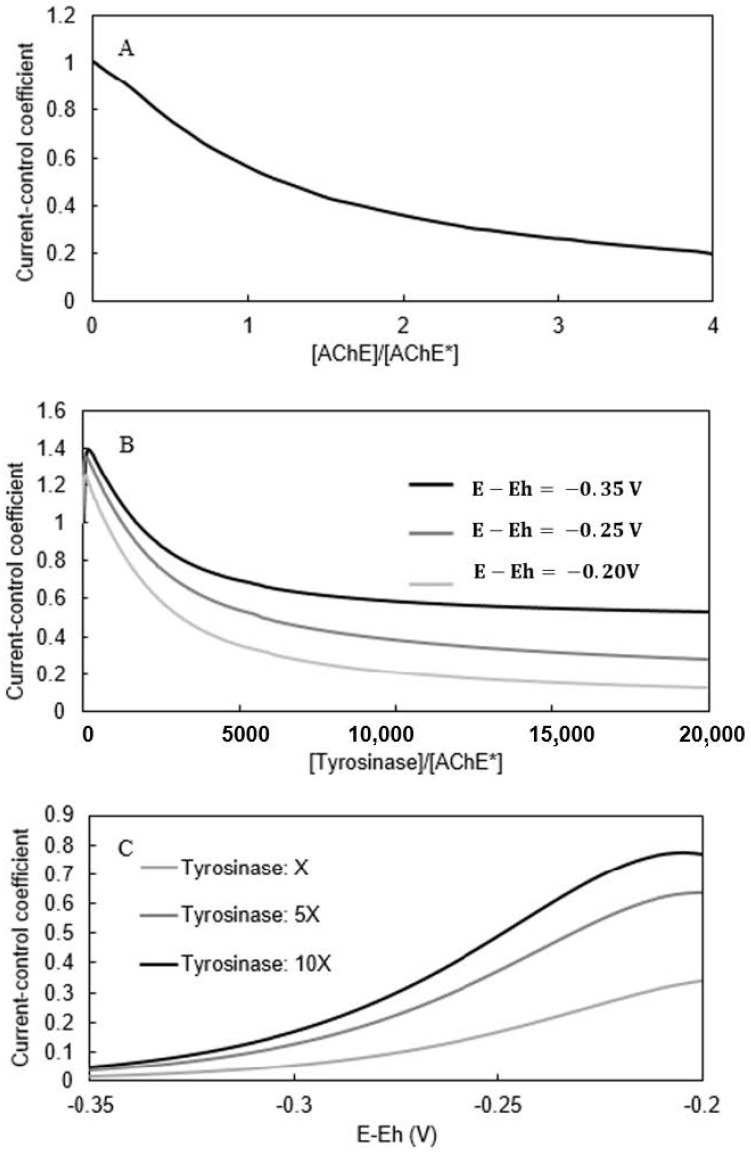
(**A**): Current-control coefficient vs. [AChE]/[AChE*]. (**B**): Current-control coefficient for tyrosinase. Tyrosinase concentration was normalized using [AChE*] = 3µM. (**C**): Current-control coefficient based on the electrochemical reaction vs. working-electrode overpotential (E-E_h_).

Flux-control analysis has been used to determine the extent to which the rates of individual enzymatic reactions limit the overall mass flux through a metabolic pathway [39]. We extended this approach to assess to what extent both individual enzymatic reactions and electrochemical reactions limited current production by the biosensor. We defined a current-control coefficient ($C_{\mathrm{Vi}}^{J})\;$for a given reaction step (V_i_) as the ratio of the percent change in the biosensor’s output current (J) to the percent change in a given V_i_ while holding all other independent variables constant (Equation (45)). We used the validated model to calculate $C_{\mathrm{Vi}}^{J}$ values for each of the three reaction rates involved in generating that current: the AChE reaction rate (V_1_), the tyrosinase reaction rate (V_2_), or the electrochemical reaction rate (V_3_). The mechanistic model allowed the enzymatic reaction rates (V_1_ and V_2_) to be varied by adjusting the assumed AChE and tyrosinase concentrations, respectively, and the electrochemical reaction rate (V_3_) to be varied by adjusting the assumed working-electrode overpotential (E-E_h_). Figure 12A–C show calculated $C_{V1}^{J}$ values as a function of the AChE concentration, $C_{V2}^{J}$ values as a function of the tyrosinase concentration, and $C_{V3}^{J}$ values as a function of overpotential (E–E_h_), respectively.

Figure 12A shows the effect of normalized AChE concentration $C_{V1}^{J}$ values. The curve declines monotonically from a value of 1 as the AChE concentration increases. For the AChE concentration used in the validated biosensor mathematical model (3 µM), a current-control coefficient of 0.52 is predicted.

(45) dJJdViVi=CViJ

Figure 12B shows the effect of tyrosinase concentration (normalized by a constant [AChE] value of 3 µM) on $C_{V2}^{J}$ values. The curve exhibits a maximum at very low tyrosinase concentrations and then declines monotonically at the tyrosinase concentration increases. Similar curve shapes were predicted for three applied working-electrode overpotentials, with $C_{\mathrm{Vi}}^{J}$ values increasing as the overpotential increases in magnitude (i.e., the working electrode becomes more negative).

Figure 12C shows the effect of applied overpotential on $C_{V3}^{J}$ values appear to decrease monotonically from a maximum value as overpotential values increase in magnitude (i.e., the working electrode becomes more negative). Similar curve shapes were predicted for the three tyrosinase concentrations studied, with $C_{V3}^{J}$ values increasing as the tyrosinase concentration increases.

Once validated, the IBE model’s predictive power has utility for guiding future biosensor design and optimization efforts. For example, Figure 8 indicates that the IBE biosensor has a sensitivity for detecting PMSF of 18.4 μA cm^−2^ (mM PMSF)^−1^. To increase that sensitivity, researchers might consider whether it would be possible to increase the percent change in biosensor output per percent change in AChE activity due to PMSF inhibition (i.e., increase $C_{V1}^{J}$). Figure 12A predicts that the $C_{V1}^{J}$ value under the experimental conditions was 0.52, and that the $C_{V1}^{J}$ value could be increased by about a factor of two by decreasing the AChE concentration. The potential utility of other strategies to increase the sensitivity could be evaluated quickly and inexpensively in silico using the model. For example, the effects of changing the thickness of the enzyme-containing layer, the concentrations and ratios of the two enzymes, the working electrode’s overpotential on the sensitivity could be rapidly assessed using the model.

## 4. Conclusions

This study demonstrated the utility of a novel experimental and modeling framework to characterize and optimize IBE electrochemical biosensors to detect markers of neurological diseases (e.g., inhibitors of neural esterases). The experimental system was an amperometric biosensor with an oxidase (tyrosinase) and a neural esterase (AChE) co-immobilized on the working electrode of a commercially available SPE array to detect markers of neurological disease. The mechanistic model included a system of coupled, partial-differential, mass-balance equations that described the simultaneous reaction and diffusion of reactants and products between the bulk solution, the enzyme-containing layer, and the working electrode. These equations, together with their boundary and initial conditions, were solved numerically using a splitting-finite-difference algorithm. The model was able to reproduce several trends in the experimental results, including a steady-state biosensor current as a function of the inhibitor (PMSF) concentration, as well as unsteady-state dynamics of the biosensor current following the addition of a reactant (phenylacetate) and an ACE inhibitor (PMSF).

The successful application of our integrated experimental and modeling framework in this paper for IBE biosensors and in a previous paper for a novel amperometric electrochemical immunosensor [26] has demonstrated that the approach is generic and has wide utility for mechanistic modeling of the key mass-transfer and reaction steps that determine the biosensor’s amplitude and sensitivity. Moreover, the novel dimensional-analysis approach (e.g., current-control coefficients, sensitivity coefficients, and Damkohler numbers) has been shown to be capable of determining the degree to which various steps limit the biosensor’s signal magnitude and sensitivity to the target analyte. These capabilities enable the framework to be used for in silico design of biosensors having performance properties that are customized for the target application, whether that might be the maximum sensitivity at low analyte concentrations or a linear response over a very wide analyte range. The framework can also predict which independent variable(s) (e.g., the thickness of the enzyme-containing layer, the concentrations and ratios of the two enzymes, the working electrode’s overpotential) would be most effective in obtaining the desired dependent performance variable(s) (e.g., signal magnitude, analyte sensitivity). Finally, this paper’s extension of the modeling capability to predict unsteady-state IBE biosensor responses provides a novel capability to design biosensors having desired dynamic properties, thereby providing the capability for a new dimension of experimental characterization using electrochemical biosensors.

## Figures and Tables

**Table 1:** Parameters and variables used in the numerical simulation.

Parameter/Variable	Dimensional Parameter	Variation Range	Value Used to Fit Experimental Data
Time	t, s	0–300	--
Distance from electrode surface	x, cm	3.0 × 10^−4^–3.0 × 10^−2^	3.0 × 10^−3^
Phenylacetate concentration	(S_1_), mM	0–1.5	0.9
PMSF concentration	(I), mM	0–0.5	--
Acetylcholinesterase concentration	(E_1_), µM	0–100	30
Tyrosinase Concentration (phenolase activity)	(E_2_), mM	0–5	1.45
Tyrosinase Concentration (catecholase activity)	(E_3_), mM	0–5	1.65
Michaelis–Menten constant of phenylacetate	$K_{m,1}$, µM	0–100	50.5
Michaelis–Menten constant of phenol	$K_{m,2}$, µM	0–10	0.25
Michaelis–Menten constant of catechol	$K_{m,3}$, µM	0–10	0.22
Acetylcholinesterase turnover number for phenylacetate	$k_{\mathrm{cat},1}$, s^−1^	2.0 × 10^2^–2.0 × 10^5^	2.3 ×10^4^
Tyrosinase turnover number for phenol	$k_{\mathrm{cat},2}$, s^−1^	2.0–2.0 × 10^3^	20
Tyrosinase turnover number for catechol	$k_{\mathrm{cat},3}$, s^−1^	2.0–2.0 × 10^3^	760
Dissociation constant of PMSF	$k_{I}$, mM	0.02–2.0	0.25
Reaction constant of deactivation of acetylcholinesterase with PMSF	$k_{2}$, s^−1^	0.001–0.1	0.005
Enzyme-containing layer thickness	L, nm	10–100	25
Diffusion layer thickness	δ, µm	10–200	30
Diffusion coefficient in diffusion layer	$D_{\delta }$, cm^2^ s^−1^	1 × 10^−6^–9 × 10^−5^	2.2 × 10^−5^
Diffusion coefficient in enzyme-containing layer	$D_{L}$, cm^2^ s^−1^	1 × 10^−8^–9 × 10^−6^	2.28 × 10^−8^
Standard redox electrochemical potential of O-quinone	$E^{0}$, V	0.15	0.15
Heterogeneous electron transfer rate constant	$K_{0}$, cm s^−1^	1 × 10^−7–^1 × 10^−4^	1 × 10^−5^

## Data Availability

Not applicable.
